# NO_*x*_ Emission Changes Over China During the COVID‐19 Epidemic Inferred From Surface NO_2_ Observations

**DOI:** 10.1029/2020GL090080

**Published:** 2020-10-02

**Authors:** Shuzhuang Feng, Fei Jiang, Hengmao Wang, Haikun Wang, Weimin Ju, Yang Shen, Yanhua Zheng, Zheng Wu, Aijun Ding

**Affiliations:** ^1^ Jiangsu Provincial Key Laboratory of Geographic Information Science and Technology, International Institute for Earth System Science Nanjing University Nanjing China; ^2^ Jiangsu Center for Collaborative Innovation in Geographical Information Resource Development and Application Nanjing China; ^3^ School of Atmospheric Sciences Nanjing University Nanjing China; ^4^ Chongqing Institute of Meteorological Sciences Chongqing China

**Keywords:** EnKF data assimilation, NO_X_ emission changes, inversion, WRF/CMAQ model, COVID‐19

## Abstract

The COVID‐19 epidemic has substantially limited human activities and affected anthropogenic emissions. In this work, daily NO_*x*_ emissions are inferred using a regional data assimilation system and hourly surface NO_2_ measurement over China. The results show that because of the coronavirus outbreak, NO_*x*_ emissions across the whole mainland China dropped sharply after 31 January, began to rise slightly in certain areas after 10 February, and gradually recover across the country after 20 February. Compared with the emissions before the outbreak, NO_*x*_ emissions fell by more than 60% and ~30% in many large cities and most small to medium cities, respectively. Overall, NO_*x*_ emissions were reduced by 36% over China, which were mainly contributed by transportation. Evaluations show that the inverted changes over eastern China are credible, whereas those in western China might be underestimated. These findings are of great significance for exploring the reduction potential of NO_*x*_ emissions in China.

## Key Points

- The spatiotemporal variations of NO_*x*_ emissions across mainland China during COVID‐19 well inferred from surface NO_2_ observations
- NO_*x*_ emissions fell by more than 60% in many large cities and by 36% across the whole mainland China due to the lockdown
- NO_*x*_ emissions dropped sharply after 31 January and began to gradually recover after 20 February across the country

## 1 Introduction

In January 2020, COVID‐19 first broke out in Hubei Province (HBP) and quickly spread across the whole China. In response to the epidemic, control measures were enhanced from HBP to the whole country with unprecedented intensity and duration. Transportation and industries were shut down and home quarantine measures were applied nationwide. These restrictions are believed to have drastically decreased air pollution emissions over China during this period, as confirmed by OMI retrievals (NASA, 2020). NO_*x*_ is mainly emitted by transportation, industry, and power plants (Li et al., 2017). Therefore, investigation of NO_*x*_ emission changes during this period could not only quantitatively evaluate the impact of the epidemic on economic activities and emission reductions over China but also provide a rare opportunity to explore the potential for emission reduction.

Generally, a “bottom‐up” method of emission inventory technology (Zhang et al., 2019) is adopted to quantify the emission changes, which depends on sector‐specific emissions factors and activity levels. Due to the temporal resolution and the lag in release of statistical data (i.e., activity level) as well as the large uncertainties in emission factors and statistical data, it is difficult to use the “bottom‐up” method to quantify short‐term and nationwide emission changes (Ding et al., 2015). Data assimilation (DA) is a “top‐down” method that can improve emissions estimates by combining observations and background fields. For example, Zhang et al. (2016) applied 4D‐VAR DA to optimize daily aerosol primary and precursor emissions over North China during the APEC 2014. Feng et al. (2020) inferred the CO emissions changes over China during the “Action Plan” using surface CO observations. Chu et al. (2018) and Ding et al. (2015) estimated PM_2.5_ and NO_*x*_ emission changes during the 2015 China Victory Day parade and the 2014 Youth Olympic Games by assimilating surface PM_2.5_ and OMI retrievals, respectively.

NO_*x*_ emission reductions during the COVID‐19 lockdown in China have been estimated based on satellite retrievals of NO_2_ (Bauwens et al., 2020; Liu et al., 2020). However, satellite retrievals usually contain large uncertainties (Deeter et al., 2015), and the estimates from different satellites might also display significant differences (Bauwens et al., 2020). Moreover, they did not consider transport and mixing of pollutants in the atmosphere, which may result in an underestimation in urban areas. In this study, a DA system is extended to infer the NO_*x*_ emissions changes over China using the nationwide surface hourly NO_2_ observations. We fully consider the mixing, transportation, and chemical reactions of pollutants in the atmosphere, as well as the relationships between the sources and acceptors. Surface observations have high accuracy, and most observations are concentrated in urban areas. Therefore, the emission changes in urban areas can be better captured in this study. A brief description of the inversion method and the prior emissions and observation data is presented in section 2. The experimental design is described in section 3. The evaluations and inverted emission changes are presented in section 4, and the conclusions are summarized in section 5.

## 2 Method and Data

### 2.1 Inversion Method

A regional CO assimilation system has been constructed and successfully applied in our previous study (Feng et al., 2020) (Feng2020), which optimized the gridded CO emissions using hourly surface CO measurements across China. In this study, the DA system was further extended to infer the gridded NO_*x*_ emissions using hourly surface NO_2_ observations over China. The system includes three components: a chemical transport model (WRF/CMAQ) (Byun & Schere, 2006; Skamarock & Klemp, 2008), and two subsystems of 3DVAR (Feng et al., 2018) and ensemble square root filter (EnSRF) (Feng et al., 2020). The WRF/CMAQ model simulates atmospheric compositions, the 3DVAR subsystem optimizes the chemical initial condition (IC), and the EnSRF subsystem infers emissions. Additional details about the three components are given in Text S1 in the supporting information.

The procedure for the inversion of NO_*x*_ emission (Figure S1) is similar to Feng2020. Before the inversion, a 5‐day and 6‐hr interval cycling assimilation using WRF/CMAQ 3DVAR framework (Jiang et al., 2013; Wu et al., 2002) is conducted to generate a “perfect” chemical IC. During the inversion, a sequential update technique is applied to optimize the emissions in view of perfect ICs. This means that after generating the optimized emissions, they are entered again into the CMAQ model to generate the initial field of the next DA window. Meanwhile, the optimized emissions are transferred to the next window as the prior emissions. In each window, the optimized emissions are calculated according to the following formulas:

(1) $$\bar{X^{a}}=\bar{X^{b}}+K\left(y-H\bar{X^{b}}\right)$$

(2) $$K=P^{b}H^{T}{\left(HP^{b}H^{T}+R\right)}^{-1}$$

where ${\bar{X}}^{b}$ and $\bar{X^{a}}$ respectively represent the prior and posterior emissions, ***H*** is observation operator, ***y*** is the observation, ***P***^***b***^ and ***R*** are background and observation error covariances, and ***K*** is Kalman gain matrix that maps the deviations in concentrations to the increments of emissions, as calculated based on ensemble simulations.

The WRF/CMAQ model is run with a simulation domain that covers the whole mainland China (WMC), which has a grid spacing of 36 km. Details on the settings can be found in Table S1 and Figure S2. It needs to be noted that the emissions are divided into point and area sources, which are located in different heights and constrained with the observations separately. The DA window is set to 1 day, and the daily mean observations are assimilated in the EnSRF algorithm. The ensemble size is set to 40, which represents a compromise between computational efficiency and assimilation performance. Covariance localization is performed to reduce spurious correlations due to sampling errors caused by the finite ensemble size. Considering the lifetime (about 10 hr, Jena et al., 2014) and wind speed (3 m/s on average, Table S2) in winter, the covariance localization radius is set to 180 km. In addition, in order to reduce the impact of the simulation deviations of the other species on the NO_*x*_ inversions, the observations of CO and SO_2_ are also simultaneously assimilated in this study.

### 2.2 Prior Emissions and Uncertainties

Within China, the anthropogenic emissions of the 2016 MEIC, and outside China, the emissions from the mosaic Asian anthropogenic emission inventory (MIX) (Li et al., 2017) are combined as the prior emission of the first DA window. The biogenic emissions are calculated offline by the Model of Emissions of Gases and Aerosols from Nature (MEGAN) (Guenther et al., 2012). The biogenic emissions are considered to be true and not optimized in this study.

As mentioned previously, the optimized emissions of the current DA window are transferred to the next DA window as the prior emissions. To avoid filter divergence, and considering compensation of the model error, prior inventory, and daily emission uncertainties, we perturb the emissions at each DA window with the same uncertainty, which is similar to the CO inversion (Feng et al., 2020). The uncertainty is set to be 25%, a value much smaller than that of CO (40%) in Feng2020 because studies (Li, Zhang, et al., 2017; Zhang et al., 2009) have shown that the uncertainty of NO_*x*_ is much smaller than that of CO in the MEIC inventory. In addition, the perturbed samples of NO_*x*_ emission are divided to NO_2_ and NO with a fixed NO_2_/NO ratio of 1/9 (Zhang et al., 2007).

### 2.3 Observation Data and Errors

The hourly averaged surface NO_2_, CO, and SO_2_ observations were obtained from the Ministry of Ecology and Environment of China (http://106.37.208.233:20035/). Altogether, there are 1,379 sites in the WMC (Figure S2). Considering the spatial representations and temporal continuity of the simulations, we deleted the NO_2_ observations that are greater than 200 μg m^−3^ or significantly larger/smaller (60 + 0.15*y(t)*, where *y(t)* represents the observations) than the data at adjacent times. The parameters were set based on experience. Overall, 0.01% of data were rejected. In addition, the NO_2_ concentrations of each city were averaged to further improve the representativeness and reduce observation error correlations (Houtekamer and Mitchell, 2001; Houtekamer and Zhang, 2016), which could avoid repeated and abnormal adjustments. Finally, observations of 336 cities were available across the WMC, from which data for 306 cities were selected for assimilation and the remaining 30 were saved for independent validation (Figure S2).

Following Feng2020, the NO_2_ observation error covariance is composed of measurement and representation errors ( $r=\sqrt{{\varepsilon_{0}}^{2}+{\varepsilon_{r}}^{2}}$). The measurement error *ε*_0_ is defined as *ε*_0_ = *ermax* + 0.005 * Π_0_, where *ermax* is a base error, which is set to 1 μg m^−3^ following Chen et al. (2019), and Π_0_ denotes the observed concentration. The representativeness error is calculated using the equations of $\varepsilon_{r}=\gamma \varepsilon_{0}\sqrt{\Delta l/L}$ (Elbern et al., 2007), where *γ* is a tunable parameter (here, γ = 0.5), Δ*l* is the grid spacing (36 km), and *L* indicates the influence radius of an observation (here, 3 km for simplification). The quality control, postprocessing and error settings of CO and SO_2_ observations are shown in Text S1.

## 3 Experimental Design

The DA system was conducted from 6 January to 29 February 2020 fully according to the procedure and settings described in section 2.1. To evaluate the posterior emissions, two parallel simulation experiments were performed using the CMAQ model, namely, a control experiment (CEP) and a validation experiment (VEP). Both experiments were run with the same model settings, WRF outputs, and initial field as the DA system. The only difference in the two experiments is the emission, where CEP is run using the combined original emission inventory as described in section 2.2, and VEP is run using the posterior emissions.

## 4 Results

### 4.1 Evaluation for Posterior NO_*x*_ Emissions

**Figure 1:**
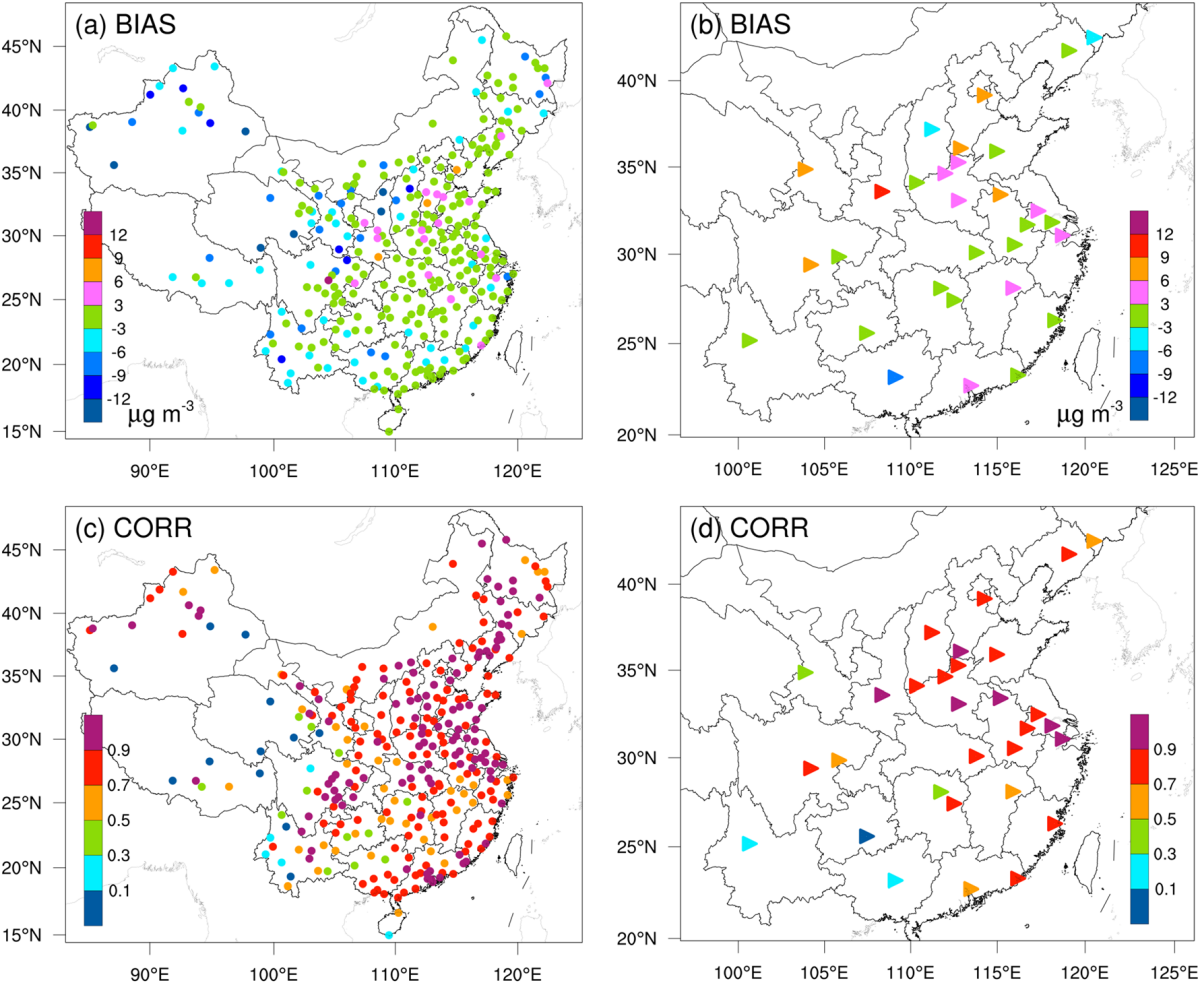
Distributions of the (a, b) mean biases (BIAS, simulated minus observed) and (c, d) correlation coefficients (CORR) of the NO_2_ concentrations simulated using the posterior emissions against the (a, c) assimilated and (b, d) independent observations, respectively.

To evaluate the overall performance of the optimized emissions, we first compared the simulated and observed meteorological factors. The evaluation results show that the WRF model satisfactorily reproduced the meteorological fields, with mean biases (BIAS) of the 10 m wind speed and 2 m temperature of 0.65 m s^−1^ and −0.06°C, respectively. These deviations in WRF simulations may result in an overestimation of NO_*x*_ emissions in North China Plain, Central China, most of East China and Northwest China, and an underestimation of emission reductions in Sichuan Basin (SCB) (Text S2 and Figure S3). Then, we indirectly validated the posterior emissions by comparing the forward simulated atmospheric concentrations against the measurements (Jiang et al., 2014). As shown in Figure 1, in most cities in eastern China, the BIAS are within ±3 μg m^−3^ and the correlation coefficients (CORR) are greater than 0.7. However, in many cities in western China, the concentrations are obviously underestimated (BIAS < −6 μg m^−3^), and the CORR are rather low (<0.3). These indicate that our system has a good performance in the inversions of NO_*x*_ emissions (Text S3) over eastern China, but still has underestimations in western China (e.g., Qinghai, Xizang, Gansu, Guangxi, and Yunnan).

### 4.2 Emission Changes During the Epidemic

**Figure 2:**
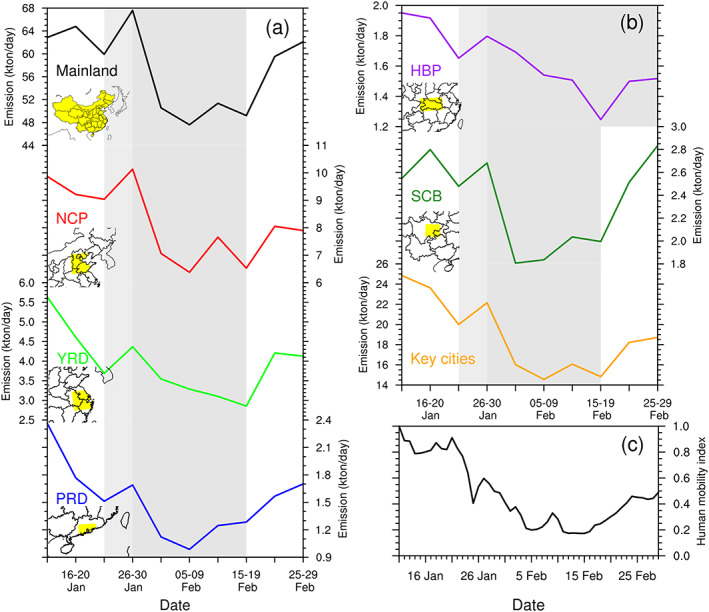
Time series of 5‐day‐averaged posterior emissions over (a) WMC, NCP, YRD, and PRD; (b) HBP, SCB, and 74 key cities; and (c) time series of normalized human mobility index of the 74 key cities (Text S4). The gray shadow indicates the lockdown period, and the light gray denotes the Spring Festival in China. The yellow shade in the small map denotes area location.

**Figure 3:**
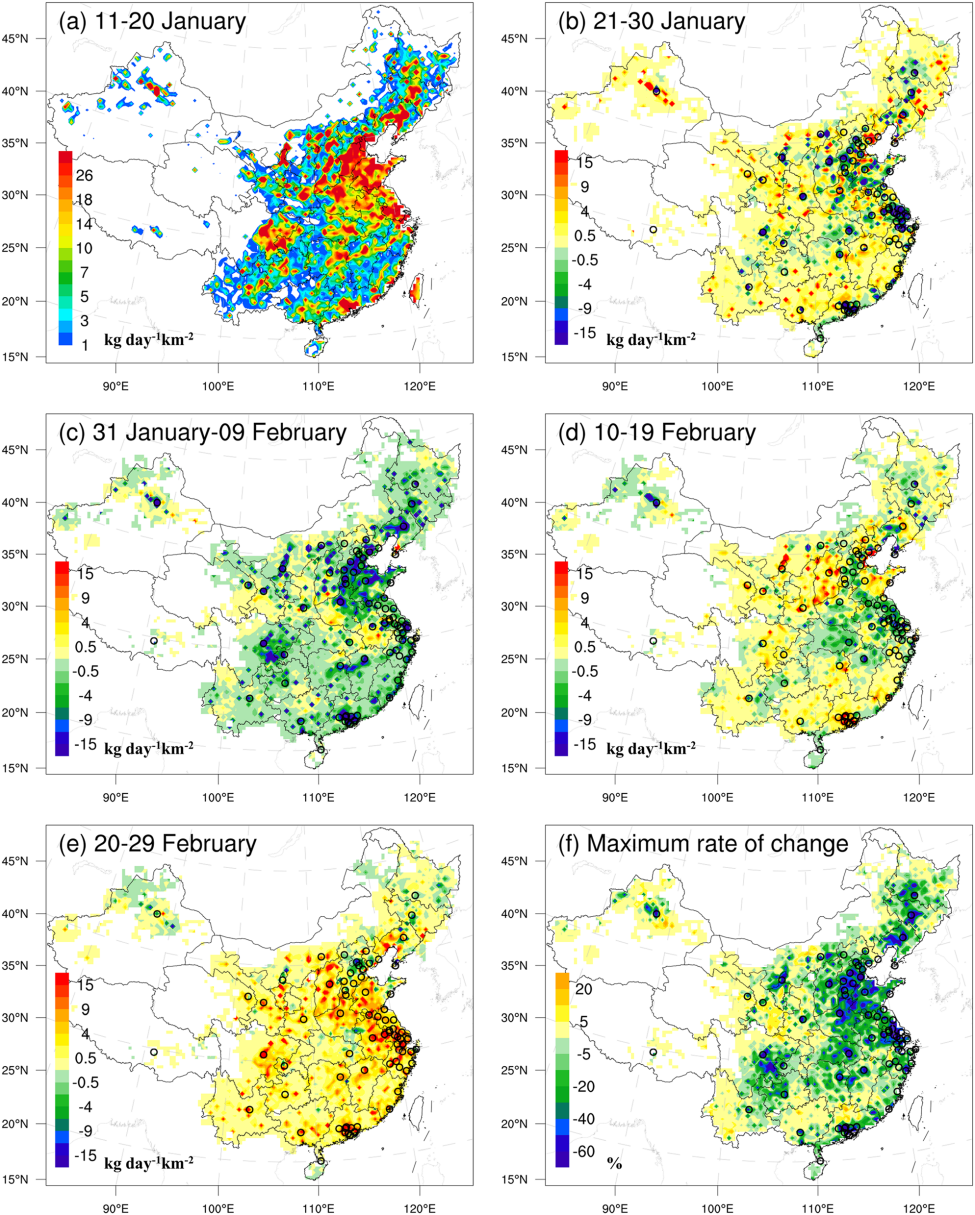
Spatial distributions of the posterior emissions, (a) averaged posterior emissions during 11–20 January, (b–e) emission changes between the current 10‐day and the previous 10‐day periods, respectively, and (f) maximum emission changes (in each grid, the value is the ratio of the minimum 10‐day moving average emission from 31 January to 20 February to the average emission during 11–20 January). Black circles denote the locations of 74 large key cities, which are the first cities that implemented the new air quality standards in 2012 in China.

To avoid the influence of model random errors (Tang et al., 2013), the 5‐day‐ or 10‐day‐averaged posterior emissions were analyzed in this study. Figure 2 shows the time series of the 5‐day‐averaged and regional mean posterior emissions for the WMC, the five key regions of the North China Plain (NCP), Yangtze River Delta (YRD), Pearl River Delta (PRD), SCB and HBP, and the 74 key cities mean for the period 11 January to 29 February 2020, and Figure 3 shows the variations of 10‐day‐averaged spatial distributions of the posterior emissions over China. Figure 2 also shows the time series of the normalized human mobility index in the 74 key cities, which can reflect the level of economic activities in the cities to a certain extent (Text S4). The locations of the five key regions and 74 key cities are shown in Figure S2.

Overall, we find two obvious processes of an initial decrease and subsequent increase in NO_*x*_ emissions during the study period. The first process occurred from 11 to 30 January, with the lowest emission during 21–25 January, and the second process occurred from 26 January to 29 February, with the lowest emissions during 1–20 February. The first process corresponds to China's Spring Festival (SF), which is the most important festival in China, that is, 24–31 January in 2020. Because of the SF, most companies started their holidays one week or 10 days before the SF, and most workers returned to rural or western areas, resulting in a gradual reduction in NO_*x*_ emissions. The increase in NO_*x*_ emissions during 26–30 January is attributed mainly to fireworks displays (Wang et al., 2007). In China, it is a traditional practice to set off fireworks frequently during the SF. Although many large cities prohibit fireworks because of air pollution, fireworks are not prohibited in most small and medium‐sized cities and all rural areas. As shown in Figure 2, the increases in NO_*x*_ emissions during the SF in PRD and the 74 key cities are much smaller than those in the others, because most areas in PRD and most of the 74 key cities prohibit fireworks. Figure 3b shows the distribution of emission changes during 21–30 January against the emissions during 11–20 January. Clearly, in most of the 74 key cities, the emissions dropped significantly, whereas in most of the remaining small and medium‐sized cities and vast rural areas, the emissions increased.

The second process is due to the coronavirus outbreak. In this process, we find that the NO_*x*_ emissions dropped sharply during 31 January to 4 February, remained at a low level from 5 February to 20 February, and gradually increased in all regions after 20 February. In fact, the outbreak in China approximately began on 23 January. On 23 January, Wuhan first began to lock down the city, and on 25 January, 30 provinces across the country announced the first‐level response to major public health emergencies. As mentioned previously, because of the fireworks during the SF, the impact of the outbreak on NO_*x*_ emissions lagged. As the SF ended, the impact of the outbreak on NO_*x*_ emissions was immediately highlighted. As shown in Figure 3c, compared with the emissions during 21–30 January, in almost all cities and most rural areas of the country, NO_*x*_ emissions decreased significantly during 31 January to 9 February. The recovery of NO_*x*_ emissions (i.e., emission increase) shows selected differences in different regions. We find that in most regions (e.g., NCP, PRD, SCB, and the 74 key cities, the mean and the entire mainland mean), the NO_*x*_ emission increased slightly after 10 February, whereas in YRD and HBP, it continued to decline until 20 February. After 20 February, as shown in Figures 2 and 3, NO_*x*_ emissions across WMC increased significantly. These indicate that production and economic activities in certain areas gradually resumed after 10 February (Wang et al., 2020), and after 20 February, they were gradually resumed basically across the WMC. These variations are in line with the time points (most are no earlier than the 10th) issued by China's local governments to allow resumption of production (Wang & Su, 2020), and are also consistent with the changes in the normalized human mobility index in the 74 key cities (Figure 2c). In addition, it is not surprising that the NO_*x*_ emissions in HBP and surrounding areas continuously decreased until 20 February, because HBP was the epidemic center of China. During 20–29 February, the average emissions over NCP, YRD, and PRD recovered to 83.6%, 81.4%, and 79.2% of the levels during 11–20 January, respectively, and in SCB, it basically recovered to the normal level. However, in HBP the relative increase was much smaller than the other regions because the HBP lockdown lasted until 25 March. The inverted emission changes over time also agree well with the variations of the satellite NO_2_ retrievals (Bauwens et al., 2020).

Taking the mean emission during 11–20 January as a reference, and the lowest 10‐day moving average of each grid from 31 January to 29 February as the emission under the lockdown, we estimate that because of the coronavirus outbreak, the NO_*x*_ emissions were reduced by more than 60% in many large cities, such as Wuhan (61%), Shenzhen (65%), Guangzhou (68%), Haerbin (66%), and by approximated 30% in most small‐ and medium‐sized cities. The rate of decrease in Beijing was estimated to be 37%. For the 74 key cities average, the emission fell by 47%. In the areas surrounding cities and in most rural areas in eastern China, the decreases were approximately 20% and 5%, respectively (Figure 3f). Instead, in certain remote areas near the Nanling and Qinling Mountains, western Yunnan, most of Qinghai, Xinjiang and Xizang provinces, where the residential emissions are dominated, the inverted emissions slightly increased (less than 5%), probably due to the return of migrant workers. In NCP, YRD, PRD, SCB, and HBP, we estimate that the largest reductions were 42%, 41%, 50%, 40%, and 35%, respectively (Table 1). For each province (Table 1), we estimate that the top five provinces with the highest NO_*x*_ emission reduction rates are Jiangsu (51%), Ningxia (51%), Jilin (50%), Liaoning (48%), and Heilongjiang (47%), and the bottom five provinces are Hainan (9%), Qinghai (10%), Xizang (15%), Chongqing (17%), and Gansu (17%). For the WMC, we estimate that the total NO_*x*_ emissions are reduced by 36%. The area and point emissions were inferred separately in this study (Text S5), and it shows that overall, the decreases in area and point emissions contributed 29% and 7% of the total 36% reduction, respectively. And for the area sources, statistics shows that the on‐road emission declined by 70% (Huang et al., 2020), and the residential emission changed little or slightly increased (Wang et al., 2020). Applying the ratio of industrial emission to traffic one from MEIC 2016, we calculated that the transport and industry sectors contributed 23% and 6% of the total reduction, respectively, and the industrial emissions decreased by 14% because of the lockdown measures. The low decline of industry is consistent with the fact that the production processes of resource‐based industries are little interrupted (Wang et al., 2015). For example, according to the Natural Bureau of Statistics data, the flat glass and crude steel emissions were estimated to be reduced by only 5.5% and 6.1%, respectively. These indicate that the reduction of NO_*x*_ emissions during the COVID‐19 lockdown is mainly from the decrease in transportation, followed by the decline of power and industrial emissions.

**Table 1:** Estimation of Regional and Provincial NO_*x*_ Emission Reductions (%) Due to Lockdown Measures Against the Coronavirus Outbreak in China

Province	Rate	Province	Rate	Province	Rate	Region	Rate
Jiangsu	−51.0	Sichuan	−38.8	Fujian	−23.1	NCP PRYRD	−41.8
Ningxia	−50.8	Xinjiang	−38.8	Guizhou	−22.3	YRD	−41.1
Jilin	−49.6	Tianjin	−38.8	Hunan	−21.3	PRD	−49.8
Liaoning	−48.1	Beijing	−37.4	Guangxi	−18.5	HBP	−34.9
Heilongjiang	−47.4	Hubei	−34.9	Yunnan	−17.9	SCB	−39.7
Guangdong	−46.2	Inner Mongolia	−33.9	Gansu	−17.0	74 cities	−47.4
Shanghai	−43.3	Henan	−30.6	Chongqing	−16.9	Mainland	−35.9
Shandong	−42.3	Anhui	−25.8	Xizang	−15.2		
Hebei	−41.6	Jiangxi	−25.4	Qinghai	−10.3		
Shanxi	−39.7	Shaanxi	−24.5	Hainan	−9.3		
Zhejiang	−39.4						

The differences in the decrease between different areas might be related not only to the lockdown measures but also to the distribution and composition of the emissions as well as the model errors. We found that the patterns of decline rates over China are similar with the distributions of NO_*x*_ emissions before the outbreak (Figure 3a). For HBP, although the reduction is relatively lower compared with the coastal developed provinces, it is the highest compared with neighboring provinces (Table 1). The large emission reduction in Ningxia is because that the emissions from power plants and big cities account for a large proportion and decreased a lot. The relatively smaller decrease in Beijing may be because the WRF/CMAQ model cannot consider the concentration increase caused by feedbacks between PM_2.5_ accumulation and boundary layer under the continuous and stable weather conditions during the lockdown (Le et al., 2020), resulting in more emissions inferred (Text S6). Bauwens et al. (2020) compared the spaceborne NO_2_ column observations over China during the COVID‐19 lockdown with those during the same period in 2019 and found that satellite NO_2_ decreased by 40% on average over Chinese cities, and by 57%, 56%, and 33% in Wuhan, Guangzhou, and Beijing, respectively. Our estimates are all slightly higher than the corresponding reductions in satellite NO_2_, which is reasonable, since due to the uneven emission reductions in different areas and atmospheric transport and mixing, the reductions in atmospheric concentrations should be smaller than the changes in emissions in the urban areas. In addition, the nationwide decrease of 36% is within the range of the “bottom‐up” estimates of the provincial NO_*x*_ reductions (29–57%) (Huang et al., 2020), and is highly consistent with the average decrease of the satellite NO_2_ over China (36%) in the week after the SF (Myllyvirta, 2020). Nevertheless, our estimates might be still slightly underestimated. Although the comparison with the emissions before the coronavirus outbreak avoids the impact of long‐term emission reductions in recent years (Shi & Brasseur, 2020), the reference emissions were weaker than the normal level, because many factories might begin their holidays 1 week or 10 days before the SF.

## 5 Conclusions

In this study, the daily NO_*x*_ emissions over China from 11 January to 29 February 2020 are optimized using a regional data assimilation system and the hourly surface NO_2_ observations across WMC. The evaluations show that in most cities in eastern China, BIAS and CORR are within ±3 μg m^−3^ and greater than 0.7, whereas in many cities in western China, significantly negative BIAS and rather low CORR occur, suggesting that the inverted changes over eastern China are credible, whereas those in western China might be underestimated.

We find that because of the coronavirus outbreak, NO_*x*_ emissions across the WMC dropped sharply after 31 January. After 10 February, NO_*x*_ emissions in certain areas began to rise slightly, and after 20 February, basically, emissions across the country began to gradually recover. The significant reductions in NO_*x*_ emissions lagged behind the lockdown, which began on 25 January, since fireworks display compensated for the reductions from lockdown during the SF. Compared with the emissions before the outbreak, NO_*x*_ emissions were reduced by more than 60% in many large cities, and by approximately 30% in most middle and small size cities. In NCP, YRD, PRD, SCB, and HBP, the largest reductions were estimated to be 42%, 41%, 50%, 40%, and 35%, respectively. Across the WMC, the NO_*x*_ emissions were reduced by 36% during the lockdown. The reductions are primarily attributed to the decrease in transportation. The contributions of power and industry emissions are comparable. This study cannot only quantitatively evaluate the impact of the epidemic on economic activities over China from the perspective of emission change but also supply a valuable benchmark for efforts to control NO_*x*_ emissions in different regions.

## Supporting information

Supporting Information S1

## Data Availability

The data used in the paper are available online (http://doi.org/10.5281/zenodo.4049744).
