# Supplementary material for: NOx Emission Changes Over China During the COVID‐19 Epidemic Inferred From Surface NO2 Observations
Source: Geophys Res Lett. 2020 Oct 2;47(19):e2020GL090080. doi: 10.1029/2020GL090080 (PMC7537042; doi:10.1029/2020GL090080)
Supplement: Supplementary file 1 — Supporting Information S1 [file GRL-47-e2020GL090080-s001.pdf]

Geophysical Research Letters

Supporting Information for

NO_x Emission Changes over China during the COVID-19 Epidemic Inferred from Surface NO_2 Observations

Shuzhuang Feng[1], Fei Jiang[1,4*], Hengmao Wang[1], Haikun Wang[2], Weimin Ju[1], Yang Shen[1], Yanhua Zheng[1], Zheng Wu[3], Aijun Ding[2]

[1] *Jiangsu Provincial Key Laboratory of Geographic Information Science and Technology, International Institute for Earth System Science, Nanjing University, Nanjing, 210023, China*

[2] *School of Atmospheric Sciences, Nanjing University, Nanjing, 210023, China*

[3] *Chongqing Institute of Meteorological Sciences， Chongqing, 401147， China*

[4] *Jiangsu Center for Collaborative Innovation in Geographical Information Resource Development and Application, Nanjing, 210023, China*

Contents of this file

1. Text S1 to S6
2. Figures S1 to S8
3. Tables S1 to S3

* Corresponding author: Tel.: +86-25-83597077; Fax: +86-25-83592288; E-mail address: jiangf@nju.edu.cn

Text S1.

WRF version 4.0 and CMAQ version 4.7.1 are applied to simulate the meteorological fields and atmospheric compositions (Byun & Schere, 2006; Skamarock & Klemp, 2008; Yu et al., 2012). WRF simulations cover the whole of East Asia (169 west-east and 129 south-north cells) with a 36-km horizontal grid spacing (Figure S2). The vertical grid on sigma-pressure coordinates was extended to 100 hPa with 51 layers. The underlying surface of urban and built-up land is replaced by the latest MODIS land cover retrieval to adapt to the rapid expansion of urban development. The CMAQ model is run with the same domain but with three gird cells removed from each side of the WRF domain. There are 15 layers in the CMAQ vertical coordinate, which are compressed from 51 WRF layers. Meteorological initial and lateral boundary conditions come from the 6-h intervals, 1° × 1° Final (FNL) Operational Global Analysis data of the National Center for Environmental Prediction (NCEP). Chemical initial and lateral boundary conditions come from the last cycle and background profiles. The Carbon Bond 05 chemical mechanism (CB05) is chosen as the gas-phase chemical mechanism (Guenther et al., 2012). Detailed physical and chemical options are listed in Table S1.

NCEP's GSI 3DVAR DA system is used to optimize chemical initial condition. To find a best estimate of analysis in the sense of minimum analysis error variance, the GSI 3DVAR algorithm considers two sources of initial information: observations at irregularly spaced points and a gridded background field. The analysis can be determined by the minimizing a scalar objective function J(x) given by

$$\mathrm{J}(\mathbf{x}_a) = \frac{1}{2}(\mathbf{x}_a - \mathbf{x}_\mathrm{b})^T\mathbf{B}^{-1}(\mathbf{x}_a - \mathbf{x}_\mathrm{b}) + \frac{1}{2}[H(\mathbf{x}_a) - \mathbf{y}]^T\mathbf{R}^{-1}[H(\mathbf{x}_a) - \mathbf{y}], \quad (1)$$

In Eq. (1), $\mathbf{x}_a$ is analysis field, $\mathbf{x}_b$ denotes the background state (3D mass concentrations), $\mathbf{y}$ is the vector of observations, H is observation operator that interpolates model variables to observation space, and **B** and **R** are the background and observation error covariance matrices, respectively, representing the relative contributions to analysis. "National Meteorological Center (NMC) method" is used to

compute the **B** by taking the differences between forecasts of different lengths valid at the common times.

EnKF is an advanced data assimilation method that features representing the background error covariance matrix by a stochastic ensemble of model state variable or parameter. It is easily implemented and can estimate the flow-dependent background error covariance matrix by simply implementing ensemble simulations without the need to additionally develop tangent linear and adjoint models (e.g., 4DVAR) that are technically difficult and cumbersome for complex chemical transport model. However, higher computational costs with ensemble simulations usually limit the resolutions of emission inventories. Spurious correlations due to sampling errors and filter divergence are also the limitations in EnKF.

In every DA cycle, the prior emissions are first perturbed to generate ensemble samples to represent error statistics (uncertainty) of the emissions. Here, we implement additive emission adjustment methods:

$$\boldsymbol{X_i^b} = \boldsymbol{X_0^b} + \delta\boldsymbol{X_i^b}, \text{ i} = 1, 2, ..., \text{N} \tag{2}$$

where $\boldsymbol{b}$ represents background state (prior emission) and $\boldsymbol{i}$ is the identifier of the perturbed samples; $\delta\boldsymbol{X_i^b}$ represents the randomly perturbed samples that are added to the prior emissions $\boldsymbol{X_0^b}$ to produce ensemble samples of the inputs $\boldsymbol{X_i^b}$. $\delta\boldsymbol{X_i^b}$ is drawn from Gaussian distributions with a zero mean and a 25% standard deviation of the prior emission in each grid. After obtaining the ensemble of state vectors, error statistics are propagated by implementing CMAQ ensemble simulations with each ensemble sample from Eq. (1) as inputs.

The EnSRF (Whitaker & Hamill, 2002), as one variant of EnKF, is a deterministic filter that obviates the need to perturb observations, which has higher computational efficiency and better performance (Sun et al., 2009). The EnSRF is formulated as follows

$$\overline{\boldsymbol{X^a}} = \overline{\boldsymbol{X^b}} + \mathbf{K}(\mathbf{y} - \boldsymbol{H}\overline{\boldsymbol{X^b}}) \tag{3}$$

$$\mathbf{K} = \boldsymbol{P^b H^T}(\boldsymbol{HP^b H^T} + \boldsymbol{R})^{-1} \tag{4}$$

$$\boldsymbol{P^b} = \frac{1}{N-1}\sum_{i=1}^{N}(\boldsymbol{X_i^b} - \overline{\boldsymbol{X}}^{\boldsymbol{b}})\,(\boldsymbol{X_i^b} - \overline{\boldsymbol{X}}^{\boldsymbol{b}})^T \tag{5}$$

where $\boldsymbol{P^b}$ is the ensemble-estimated background error covariance matrix, $\boldsymbol{K}$ is the Kalman gain matrix, and N is the ensemble size. Combined with observational vector $\boldsymbol{y}$, the ensemble mean $\overline{\boldsymbol{X^a}}$ of the analysis state are updated.

The quality control method of CO and SO_2 employed is similar to that of NO_2. CO and SO_2 values larger than 10 mg m^{-3} and 400 μg m^{-3} respectively are classified as unrealistic and rejected. Additionally, time-continuity is checked to eliminate the values larger/smaller ($Ta + 0.15y(t)$) than the data at adjacent times, where $y(t)$ represents the observations, and Ta is set to 2 and 80 for CO and SO_2, respectively. Observations within each city and the same grid are averaged to improve the representativeness and reduced the observation error correlations. For error settings, except that the $ermax$ of measurement error is set to 0.02 and 1.0 for CO and SO_2, respectively, other parameters are the same as those of NO_2.

Text S2.

The performance of meteorological simulation is critical for emission inversion, because meteorological processes notably affect pollutants' transport, mixing and chemical reactions, and determine the estimation of the flow-dependent background error covariance. Generally, both higher temperature and lower relative humidity lead to a faster photolysis of NO_2, and a stronger wind corresponds to a better diffusion and transport of air pollutants, resulting in lower NO_2 concentrations in the atmosphere. Because all the biases between the simulated and observed concentrations are assumed to be attributed to the emissions during the inversion, the lower concentrations caused by the errors in the simulated meteorological fields may lead to overestimation of the emissions. To quantitatively evaluate the accuracy of the simulated meteorological fields, the mean bias (BIAS), root mean square error (RMSE), and correlation coefficient (CORR) are calculated against the surface meteorological observations

obtained from the National Climate Data Center (NCDC) integrated surface database (http://www.ncdc.noaa.gov/oa/ncdc.html). The spatial distribution of 350 meteorological sites is shown in Figure S2. The simulations were conducted from 11 January to 29 February 2020. Figure S3 shows the spatial distribution of the mean bias of the WRF simulations and their changes before and during the outbreak. The simulated wind speeds at 10 m (WS10) are overestimated nationwide, with biases in most sites in range of 0.3 to 0.9 m/s. For the temperature at 2 m (T2), there are positive biases in North China Plain, Central China, most of East China and Northwest China, and negative deviations in the rest areas. The performance of relative humidity at 2 m (RH2) is just the opposite of T2. These indicate that the emissions over North China Plain, Central China, most of East China and Northwest China may be overestimated because of the deviations in WRF simulations, while in Northeast and Southwest China, the underestimated T2 and overestimated RH2 might result in an underestimate in emission inversion, but the overestimated WS10 may offset this impact. Figure S3d-e shows the differences of meteorological biases before and during the lockdown (during minus before). Except for Sichuan Basin (SCB), the differences in the biases of WS10 simulations are small before and during the outbreak. In SCB, the biases of wind speed during the outbreak are increased, meanwhile, the biases of T2 are also increased, suggesting that the estimated reductions of NO_x emissions in SCB may be underestimated. Table S2 summarizes the statistical results of the evaluations of the simulated meteorological parameters averaged over all 350 stations. Overall, the T2 and RH2 are slightly underestimated and WS10 is overestimated, with biases of -0.06 °C, -1.43% and 0.65 m/s, respectively. Although there are relatively large biases for the simulations of WS10, it is better than other studies (Chen et al., 2016; Jiang et al., 2012a; Jiang et al., 2012b). The CORRs are approximately 0.99 for T2 and 0.96 for RH2, showing good consistency between observations and simulations. Therefore, the WRF can generally well reproduce the meteorological conditions, which is adequate for our inversion estimation.

Text S3.

Figure S5 shows the daily mean spatial distributions of the prior and posterior NO_x emissions and their differences. Although the posterior NO_x emissions changed heterogeneously in different regions, the spatial distributions are similar in general, with higher emissions mainly in the North China Plain (NCP), Yangtze River Delta (YRD) and Pearl River Delta (PRD) regions and lower emissions across Northeast, Northwest and Southwest China.

Compared with the prior emissions, significant decreases mainly occurred in NCP, YRD and parts of Central China, which may be mainly attributed to the China's clean air actions (Zheng et al., 2018). For other regions, emissions mainly exhibit moderate increases. Noted that the emission changes in or around the city are larger, which usually consistent with the distribution of major contribution sectors (e.g. industry, transportation and power plants) of NO_x. The differences between the two sets of emissions reflect the joint influence of the deficiencies of the prior emissions and the changes between two stages. Overall, statistics show that the emissions generally are decreased over mainland China with the total NO_x emissions decreased to 44.4 kton/day, 3.7% lower than the prior emissions, which mainly contributed by decreases of those in the NCP, YRD, PRD and Hubei Province (HBP) with decreases of 27.5%, 41.6%, 16.4% and 25.6%, respectively. Although the difference of the total emissions across the country between prior and posterior emissions is small, the difference at the regional/local scale is quite large (Figure S5d).

Figure S6 shows the time series of the simulated and observed daily NO_2 concentrations in NCP, YRD, PRD, HBP and SCB. Clearly, the concentrations simulated using the posterior emissions are highly consistent with the observations in all regions. The statistics show that compared against the assimilated observations, the BIAS are in the range of -0.7 to 1.9 μg m^{-3} and the CORR are in the range of 0.84 to 0.91. Compared with the independent observations, the simulations also have good performance, with BIAS and CORR in the range of -0.8 to 6.0 μg m^{-3} and 0.69 to 0.89, respectively, in

different regions (Table S3).

Text S4.

The city human mobility index represents the scale of the population moving into the city. The administrative boundary is adopted as the migration boundary of the city. Therefore, the index could reflect the human activity level in a specific city to some extent. These migration data were obtained from the Baidu Map (http://qianxi.baidu.com/), which uses big data technology to analyze "location-based services" data, and dynamically and real-time displays the source and destination of population migration from the regional and time dimensions.

Text S5.

Figure S7 shows the spatial distributions of the point emission changes, which is basically consistent with the total emission change. Overall, the point emissions also fell the most between January 31 and February 9. In mainland China, NCP, YRD, PRD, HBP, and SCB, point emissions are reduced by 35%, 35%, 29%, 30%, 32%, 20%, respectively.

Text S6.

Figure S8 showed a sensitivity simulation to evaluate the impact of different weather conditions on NO_2 concentrations in Beijing, which was simulated using the meteorological fields in the same periods in 2019. Comparing the meteorological factors between the same period in 2020 and 2019 in Beijing shows that the temperature at 2 m (T2) and relative humidity at 2 m (RH2) in 2020 are much higher than those in 2019, with positive deviations of 1.0 ℃ and 20.0%, while the wind speeds at 10 m (WS10) is much weaker than the one in 2019, with negative deviation of -0.3 m/s, indicating that the weather conditions in Beijing in 2020 are more stable than those in the same period in 2019. The sensitivity simulation shows that the simulated concentrations with meteorological fields in 2020 are 5.4 ug/m^3 (22.3%) higher than that simulated in 2019 (Figure S8), implying that the emissions reductions directly

estimated based on the changes in atmospheric concentrations like satellite retrievals may be significantly underestimated.

Additionally, the NO_x emissions were reduced most significantly during February 5 - 14 in NCP. Severe $PM_{2.5}$ pollution in Beijing, with the maximum hourly concentration exceeding 250 ug/m^3, also occurred in this period. Compared with the $PM_{2.5}$ concentrations before the epidemic (January 11 - 20, the same thereafter) (59.8 ug/m^3), the mean concentration (108.3 ug/m^3) during this period was almost twice as high as before. Severe $PM_{2.5}$ concentrations would strengthen the feedbacks between meteorological conditions and air pollutions, and change the regional and local weather conditions, so as to increase the biases between the simulated and observed meteorological factors (Ding et al., 2013). The WRF-CMAQ model used in this study is an off-line air quality model, which means that the feedbacks between air pollution and weather conditions can not be considered in this study. Comparing the simulation biases of meteorological factors during (February 5 - 14, the same thereafter) and before the epidemic shows that before the epidemic, the biases of WS10, T2 and RH2 were 0.15 m/s, 0.31 °C and -8.30 %, respectively, and during the epidemic, the biases of WS10, T2 and RH2 were significantly increased, with biases of 0.39 m/s, 1.91 °C and -17.82%, respectively. Specifically, the maximum daily average temperature bias reached up to 3.5 °C. These indicate that the simulation biases of the WRF model were significantly increased during the epidemic. The enhanced feedback between the meteorology and air pollutions in Beijing during the epidemic is also proved by Le et al. (2020). Usually, a higher temperature and lower relative humidity lead to a faster oxidation of NO_2, and a larger wind speed corresponds to better diffusion and transport of air pollutants, all resulting in lower NO_2 concentrations in the atmosphere, therefore, the increases in meteorological simulation error would lead to lower simulated NO_2 concentrations during the epidemic, resulting in more emissions were inferred to compensate this underestimation, and accordingly, the emission reduction in Beijing was underestimated in this study.

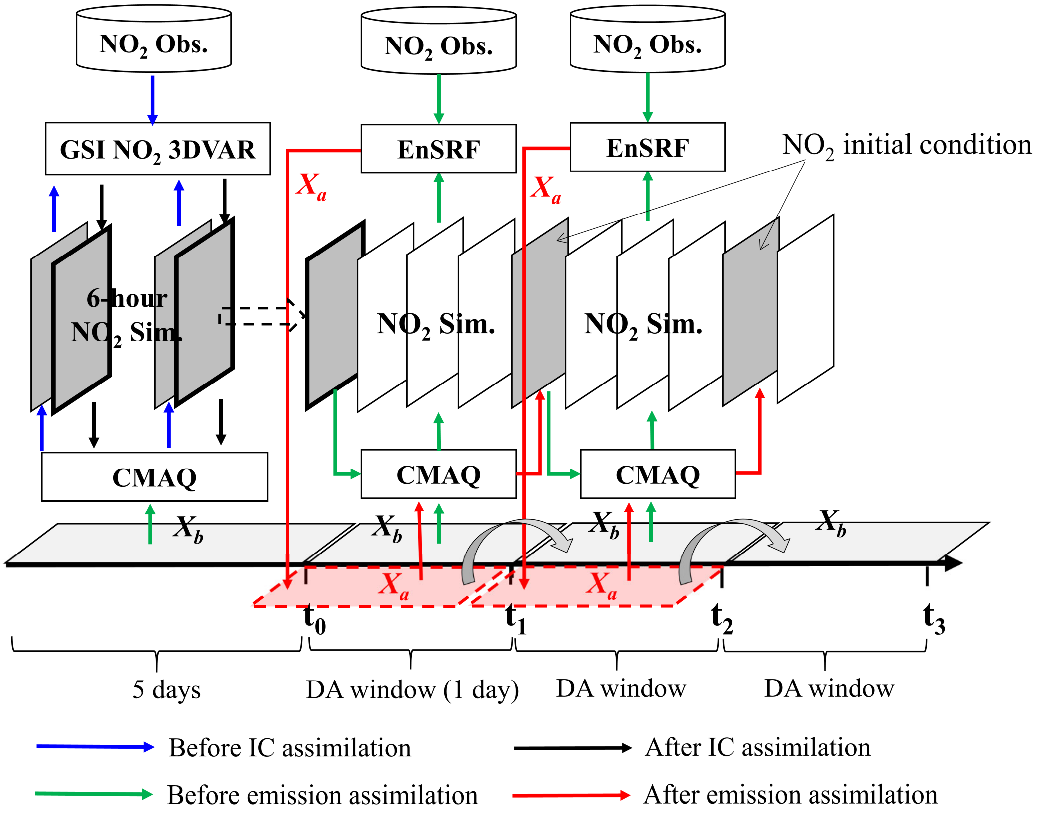

Figure S1. Flow chart of the cycling assimilations in this study.

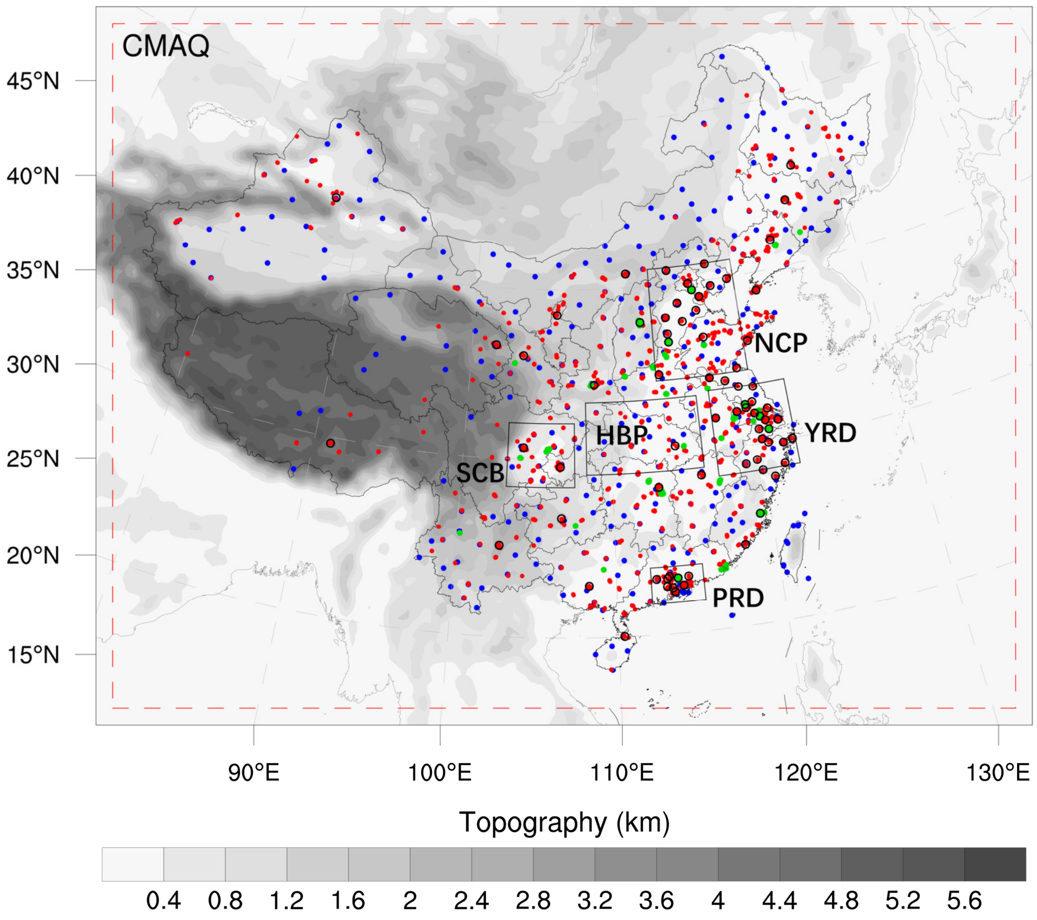

Figure S2. Model domain and observation network. The red dashed frame depicts the CMAQ computational domain; the red and green dots are the measurement sites located in assimilated and independent evaluation cities, respectively; the blue dots are the meteorological measurement sites; the black circles are the locations of 74 large key cities; the five boxed subregions are the North China Plain (NCP), Yangtze River Delta (YRD), Pearl River Delta (PRD), Hubei Province (HBP) and Sichuan Basin (SCB); and finally, the shaded area depicts the topography.

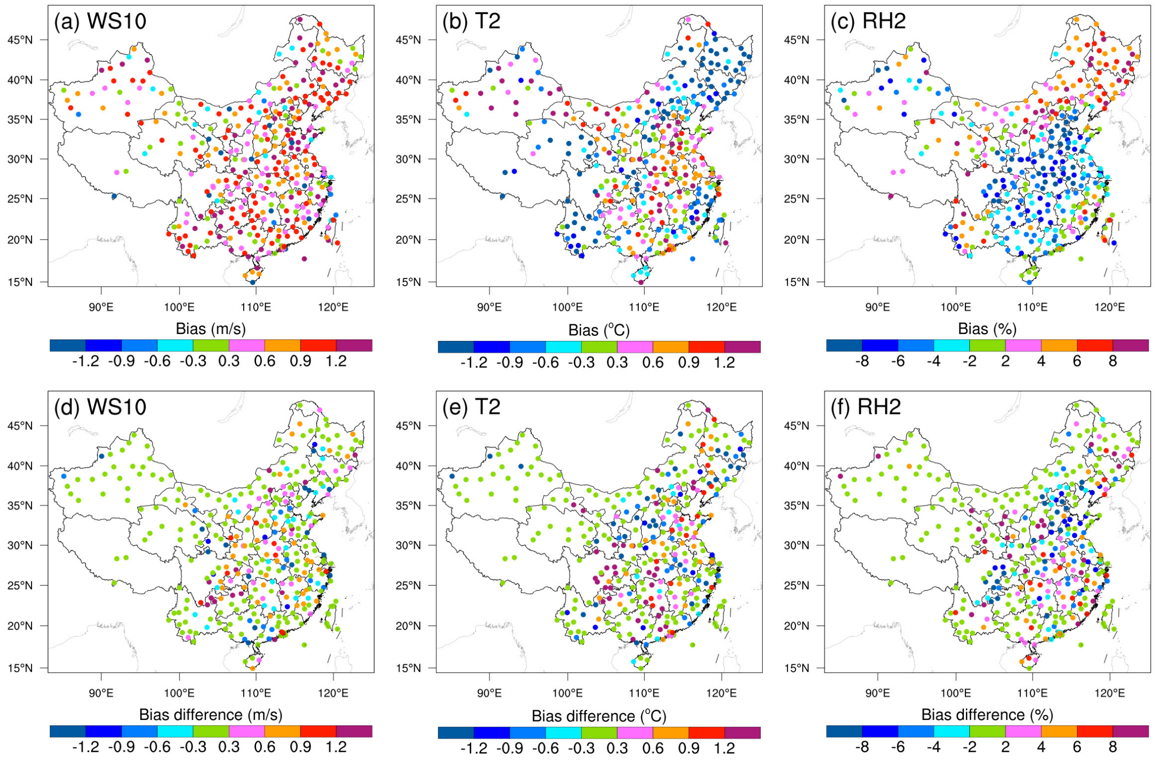

Figure S3. Spatial distribution of the (a-c) mean biases of the simulated temperature at 2m (T2), relative humidity at 2 m (RH2) and wind speed at 10 m (WS10), and the (d-e) differences in the meteorological biases between the days before lockdown (11-20 January) and the days with the maximum emission changes during the COVID-19 lockdown.

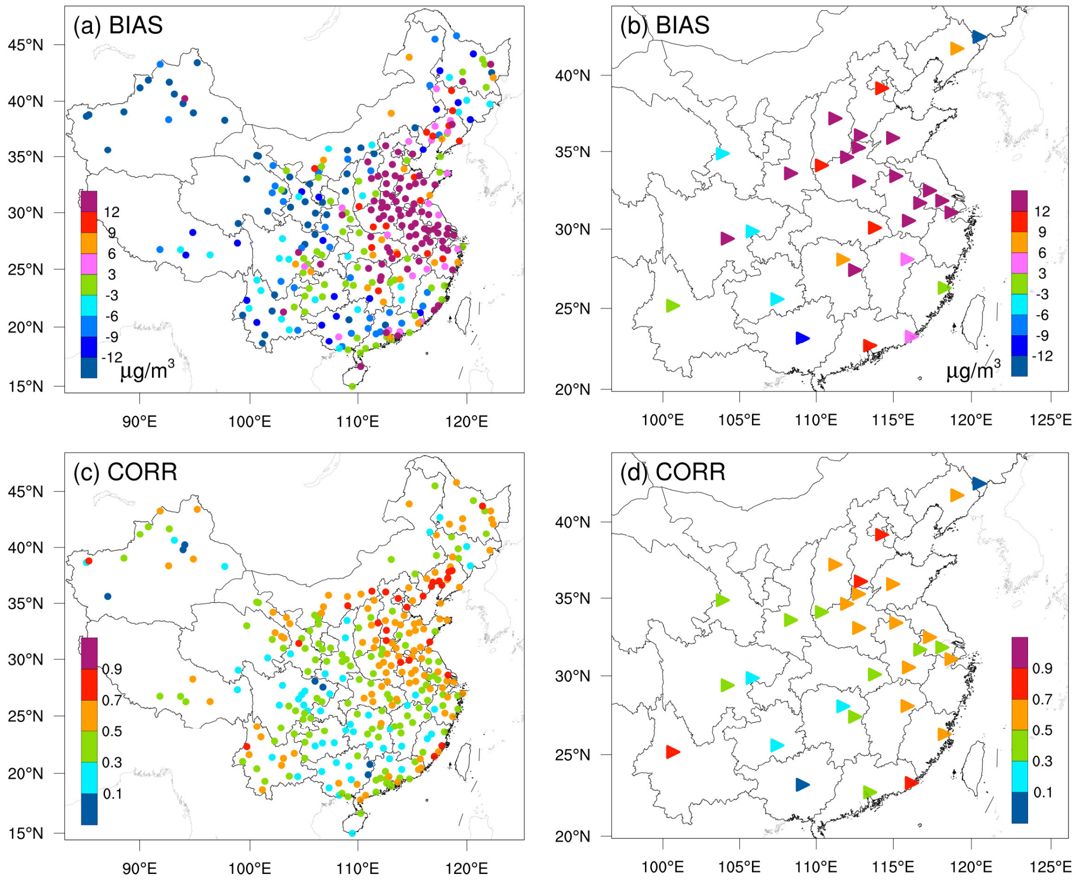

Figure S4. Distributions of the (a, b) mean biases (BIAS, simulated minus observed) and (c, d) correlation coefficients (CORR) of the NO_2 concentrations simulated using the prior emissions against the (a, c) assimilated and (b, d) independent observations, respectively.

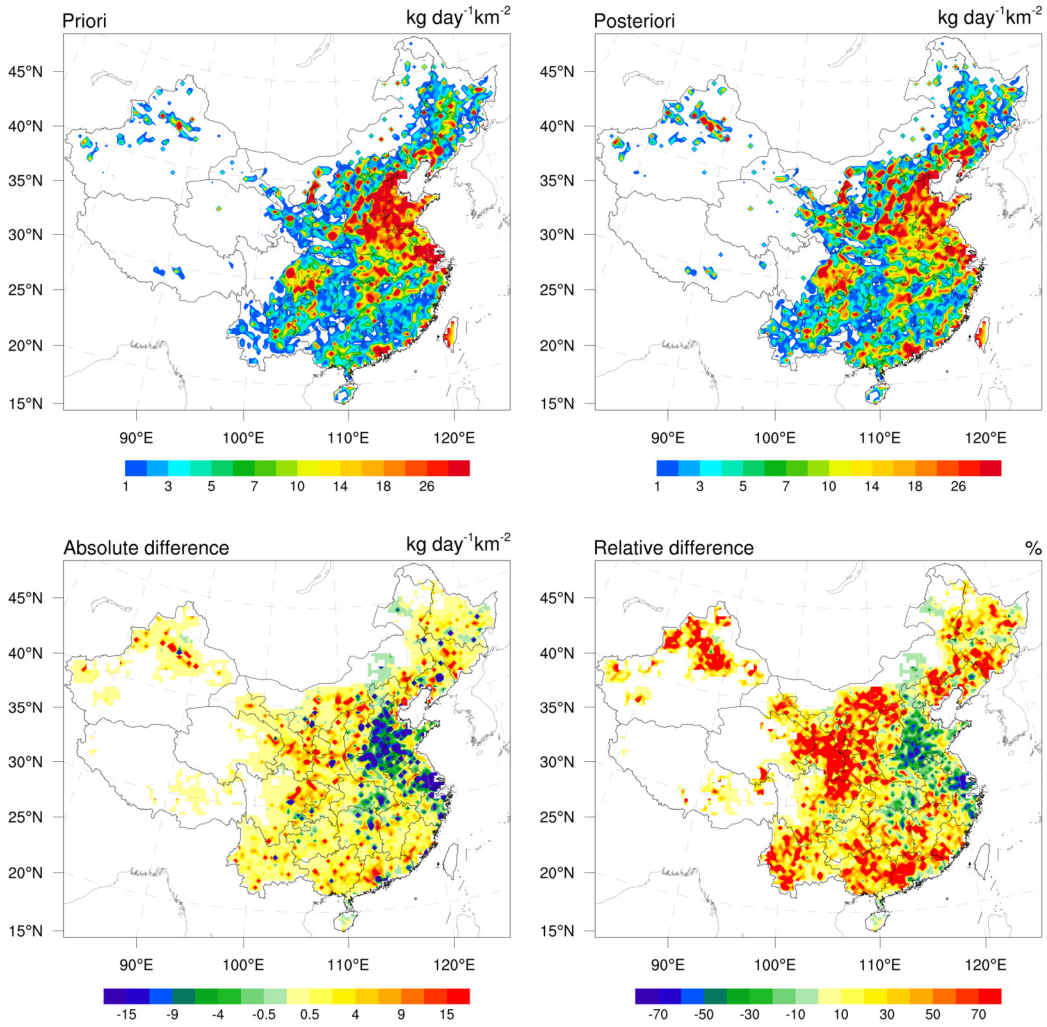

Figure S5. Spatial distribution of (a) prior emissions, (b) posterior emissions, (c) absolute differences (posterior emission minus prior emissions, kg·day^{-1}km^{-2}), and (d) relative difference (%).

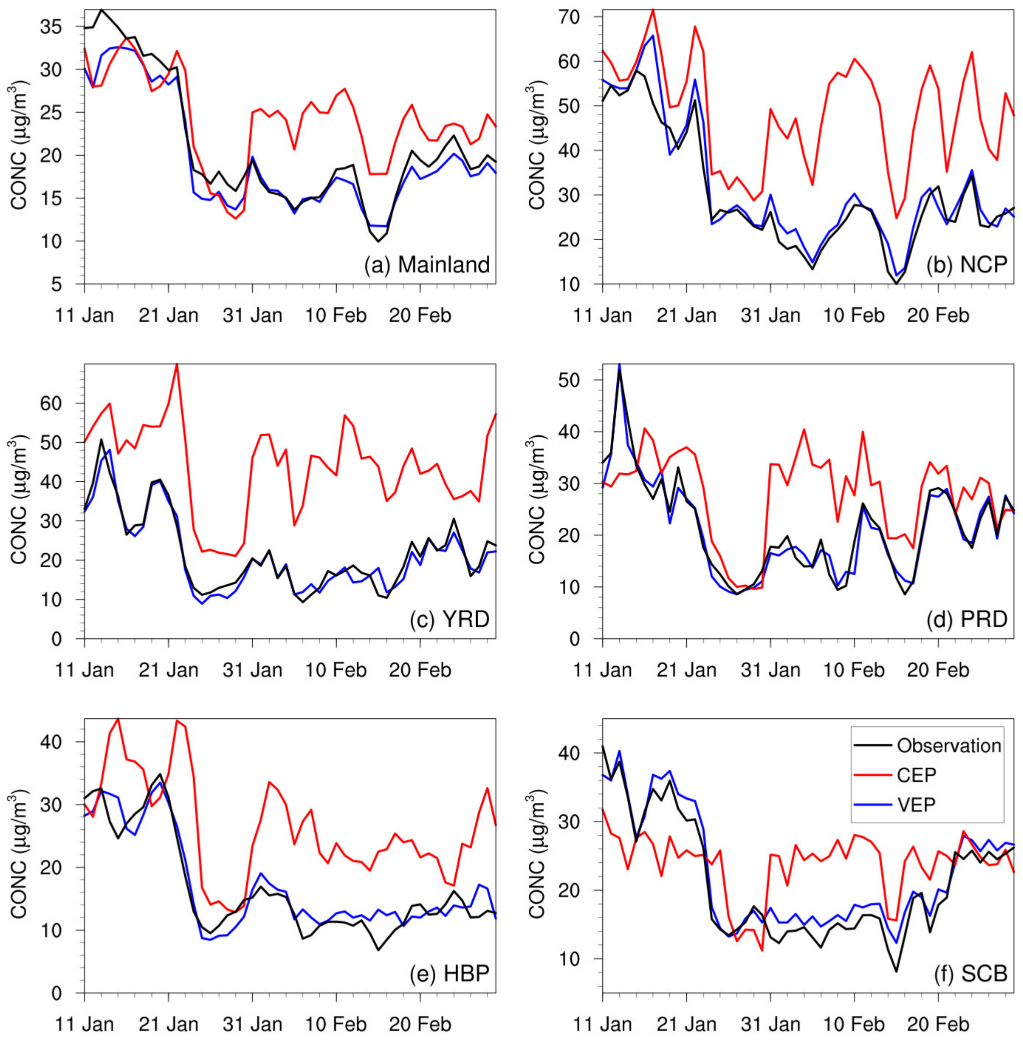

Figure S6. Time series of the daily NO_2 concentrations (CONC) averaged over the (a) Mainland, (b) NCP, (c) YRD, (d) PRD, (e) HBP and (f) SCB obtained from the observations, control experiment (CEP) and validation experiment (VEP).

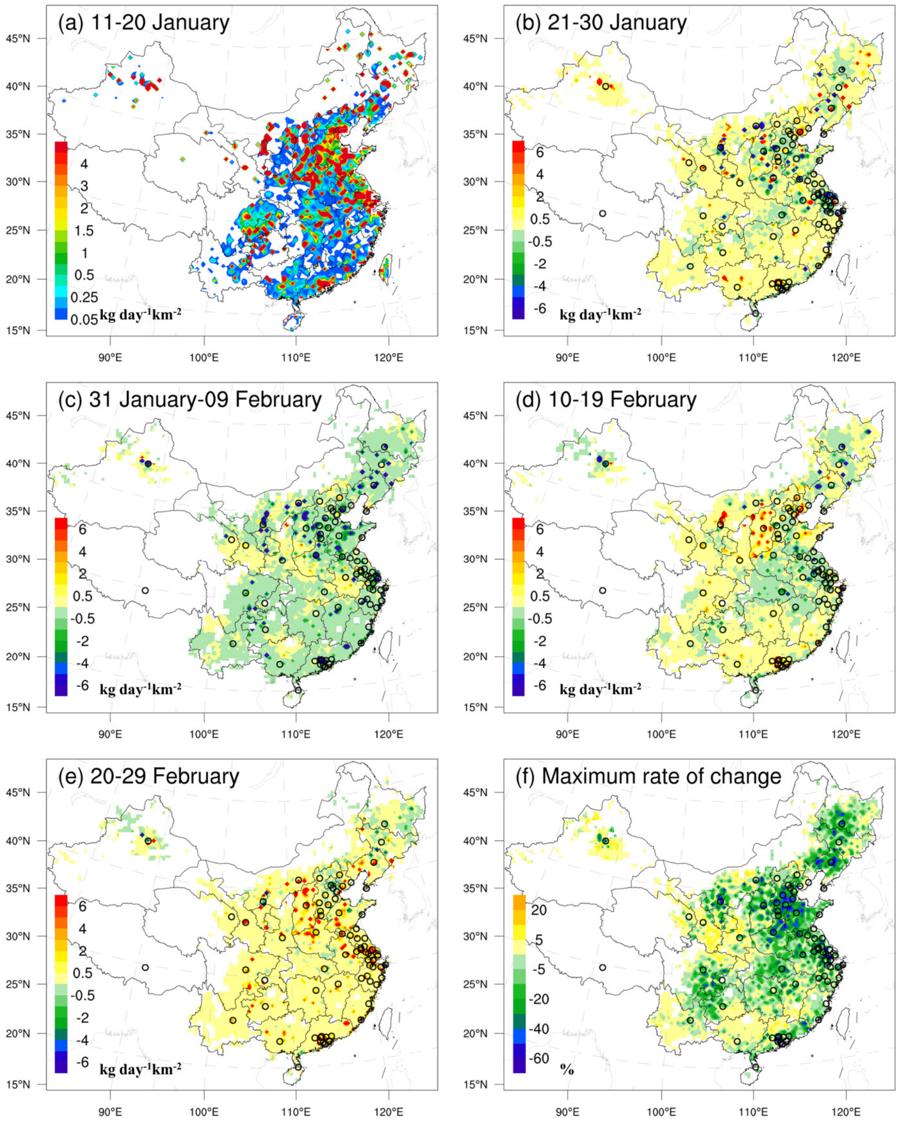

Figure S7. Spatial distributions of the posterior point emissions, (a) averaged posterior emissions during 11-20 January, (b-e) emission changes between the current 10-day and the previous 10-day periods, respectively, and (f) maximum emission changes (in each grid, the value is the ratio of the minimum 10-day moving average emission from 31 January to 20 February to the average emission during 11-20 January). Black circles denote the locations of 74 large key cities, which are the first cities that implemented the new air quality standards in 2012 in China.

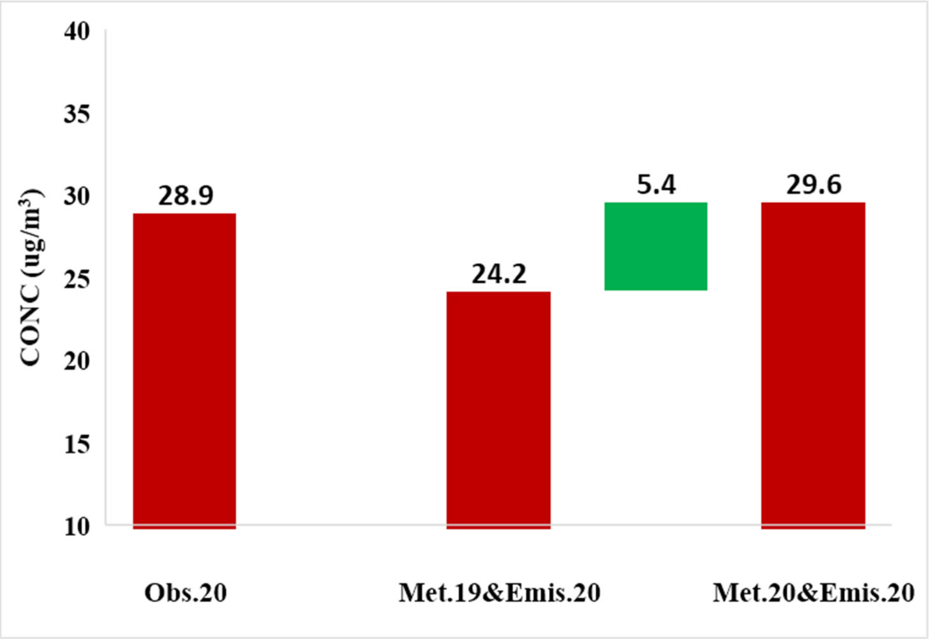

Figure S8. The observed and simulated NO_2 concentration in Beijing during the study period. The simulations are driven by meteorological fields in 2019 and 2020, respectively, and the same emissions inferred in this study.

Table S1. Configuration options of WRF/CMAQ

WRF		CMAQ	
Parameter	Scheme	Parameter	Scheme
Microphysics	WSM6	Horizontal/Vertical	yamo/yamo
Longwave	RRTM	Horizontal/Vertical	multiscale/acm2_inli
Shortwave	Goddard	Deposition	aero_depv2
Boundary layer	ACM	Chemistry solver	EBI
Cumulus	Kain-Fritsch	Photolysis	table
Land-surface	Noah	Aerosol module	AERO4
Surface layer	Revised	Cloud module	cloud_acm
Urban canopy	No	Gas-phase chemistry	CB05cl

Table S2. Statistics comparing the simulated and observed 10-m wind speed (WS10, m/s), 2-m temperature (T2, °C), and 2-m relative humidity (RH2, %) averaged over all 350 stations.

Variable Met.	Mean Obs.	Mean Sim.	BIAS	RMSE	CORR
WS10 (m/s)	2.40	3.05	0.65	0.76	0.73
T2 (°C)	2.22	2.15	-0.07	0.69	0.99
RH2 (%)	67.95	66.52	-1.43	3.52	0.96

Table S3. Statistics comparing the NO_2 concentrations from the simulations with prior and posterior emissions against assimilated and independent observations, respectively. Statistics are calculated against daily regional averaged observations (50 pairs). The number next to the region name indicates the number of cities.

Region	Mean Obs. (μg m^{-3})	Mean Sim. (μg m^{-3})		BIAS* (μg m^{-3})		RMSE* (μg m^{-3})		CORR*	
		CEP	VEP	CEP	VEP	CEP	VEP	CEP	VEP
Against assimilated observations									
Mainland (306)	21.1	24.2	19.7	3.1	-1.4	19.2	7.1	0.49	0.88
NCP (31)	29.8	48.4	31.8	18.6	1.9	25.2	7.7	0.56	0.91
YRD (24)	21.9	43.7	21.2	21.9	-0.7	29.8	6.3	0.52	0.88
PRD (9)	21.3	28.0	21.0	6.6	-0.4	20.7	5.5	0.27	0.89
HBP (20)	16.6	26.4	17.1	9.8	0.5	17.5	5.3	0.53	0.87
SCB (14)	21.2	23.9	22.6	2.8	1.4	18.8	6.7	0.29	0.84
Against independent observations									
Mainland (30)	22.8	34.3	25.1	11.5	2.4	21.0	8.8	0.60	0.84
NCP (4)	31.9	50.3	37.9	18.4	6.0	22.5	10.2	0.68	0.89
YRD (5)	23.5	47.4	25.5	23.9	1.9	29.5	6.7	0.53	0.87
PRD (1)	20.6	30.4	24.2	9.8	3.6	17.4	9.5	0.40	0.69
HBP (1)	18.0	27.5	17.2	9.5	-0.8	14.8	5.2	0.41	0.76
SCB (2)	18.8	25.0	21.8	6.2	3.0	16.0	8.1	0.39	0.76

* BIAS, mean bias; RMSE, root mean square error; CORR, correlation coefficient
